# Effectiveness of Physiotherapy Interventions in Pleural Effusion Patients: A Comprehensive Review

**DOI:** 10.7759/cureus.61195

**Published:** 2024-05-27

**Authors:** Saurabh Zunzunwala, Pratik R Jaiswal

**Affiliations:** 1 Cardiovascular and Respiratory Physiotherapy, Ravi Nair Physiotherapy College, Datta Meghe Institute of Higher Education & Research, Wardha, IND; 2 Sports Physiotherapy, Ravi Nair Physiotherapy College, Datta Meghe Institute of Higher Education & Research, Wardha, IND

**Keywords:** pulmonary function, chest expansion, intercostal drainage, chest physiotherapy, pleural effusion

## Abstract

Pleural effusion, characterized by the accumulation of fluid between the parietal and visceral pleura, presents significant challenges in patient management, particularly in cases of malignant pleural effusion. Despite various therapeutic options, there is a need to evaluate the effectiveness of physiotherapy interventions specifically for pleural effusion patients, as current literature predominantly focuses on medical and surgical treatments. This comprehensive review aims to address this research gap by systematically analyzing the impact of physiotherapy on pleural effusion management, with a focus on symptom relief and improvement in quality of life. The objective is to determine the role of physiotherapy in reducing hospital stay and enhancing patient outcomes. Methodologically, this review synthesizes data from clinical studies and case reports that document physiotherapy interventions, such as breathing exercises, postural drainage, and mobilization techniques, in the treatment of pleural effusion. Our findings suggest that physiotherapy interventions can significantly alleviate dyspnoea and improve respiratory function, contributing to better overall patient outcomes. These results underscore the importance of incorporating physiotherapy into the standard care protocol for patients presenting with pleural effusion to optimize recovery and quality of life.

## Introduction and background

Pleural effusion is the accumulation of fluid in the layers of the pleura. Even though pleural illnesses are frequently indicated by the symptoms (shortness of breath, chest pain, cough) that patients complain of, diagnosing pleural effusion is quite challenging. Pleural effusion can arise from different aetiologies and is defined by the pleural cavity being filled with transudative or exudative pleural fluids. Radiological investigations such as computed tomography, ultrasonography, or basic chest radiography can be used to confirm the existence of pleural effusion. Appropriate treatment for pleural effusions requires the investigation of pleural fluid to determine the aetiology of the effusions [1]. For patients with pleural effusion and chest drains, conventional chest physiotherapy and intermittent positive airway pressure breathing are commonly recommended [2]. Conventional therapy can also include exercises such as breathing exercises, belt exercises, localized expansion exercises, positioning, and others that are part of the physiotherapy management of pleural effusion. Exercises for enhancing chest wall, trunk, and shoulder mobility are beneficial since they also increase airflow on that side of the chest, highlight the depth of perspiration, and regulate expiration. Subjects with pleural effusion can improve their chest expansion with these activities [3]. Combining respiratory physiotherapy, which includes breathing exercises, posture correction exercises and mobilizations, sputum clearance exercises, and patient education, with medical treatment and drainage for pleural effusion led to a reduction in the length of hospital stay and an improvement in recovery. Changes in intrathoracic pressure brought on by this combo therapy enhance expansion by improving drainage [4].

Conventional treatment can also include postural drainage, which has been crucial in raising lung volumes, perfusion, oxygenation, and secretion mobilization. While postural drainage primarily assists in managing respiratory secretions rather than directly reducing pleural effusion, it helps improve overall lung function and respiratory efficiency. Gravity affects perfusion, lymphatic drainage, ventilation depth, and pattern, which collectively support better breathing mechanics and patient comfort. For a wide variety of individuals, rotating or shifting positions frequently is an effective way to preserve lung health [5]. When high trachea bronchial secretions characterize a condition, postural drainage improves mucociliary clearance and results in larger sputum quantities than a comparable control period [6]. A tool for training respiratory muscles is called lung boost. Several studies have shown that combining a fitness routine like walking with a respiratory muscle training device strengthens and extends the respiratory muscles. As a result, additional benefits may include increased walking distance, decreased dyspnoea, enhanced quality of life, and an overall sense of well-being. Lung boost is intended for people with pleural effusion as well as anyone who wants to increase their own power of respiratory muscles and endurance [7]. Around the world, malignant pleural effusion (MPE) is a common condition with high morbidity and fatality rates. An estimated 150,000 Americans and over 100,000 Europeans are impacted by MPE annually [8]. In comparison to patients without pleural effusion, those with pleural effusion had a significantly higher in-hospital mortality rate (9.9%) and a longer hospital stay [9].

MPE is a common clinical syndrome that arises in cancer patients with many types of malignancies; its presence signals the beginning of the malignancy's terminal phases [10]. Lymphomas cause 10% of MPE cases. Effusion can be observed in non-Hodgkin's lymphoma as early as the time of diagnosis, but it can be observed in Hodgkin's lymphoma during the later stages of the disease's course [11]. Positive airway pressure breathing and incentive spirometry contribute to lung expansion therapy. By raising the transpulmonary pressure gradient, incentive spirometry is an essential component of lung expansion therapy. In incentive spirometry, sustained maximum inspiration is the basic manoeuvre. A slowed, deep inhalation is a breathing technique used primarily to take precautions to prevent lungs from collapsing and atelectasis in postoperative patients. It involves slowly filling the lung to the full volume, followed by a 5- to 10-second breath hold [10]. Thoracentesis, a technique that consists of aspirating the fluid with an intercostal needle, is one way to manage some pleural fluid collections. The British Thoracic Society guidelines advise the insertion of an intercostal tube connected to a closed drainage system in cases of MPE, empyema, traumatic hemopneumothorax, and following thoracic surgery [12].

This review aims to check the effectiveness of physiotherapy protocol, mainly in pleural effusion patients. It also covers the recent advances that are ongoing in the physiotherapy treatment of pleural effusion patients and how it is useful for treating those patients.

## Review

Search strategy

**Figure 1:**
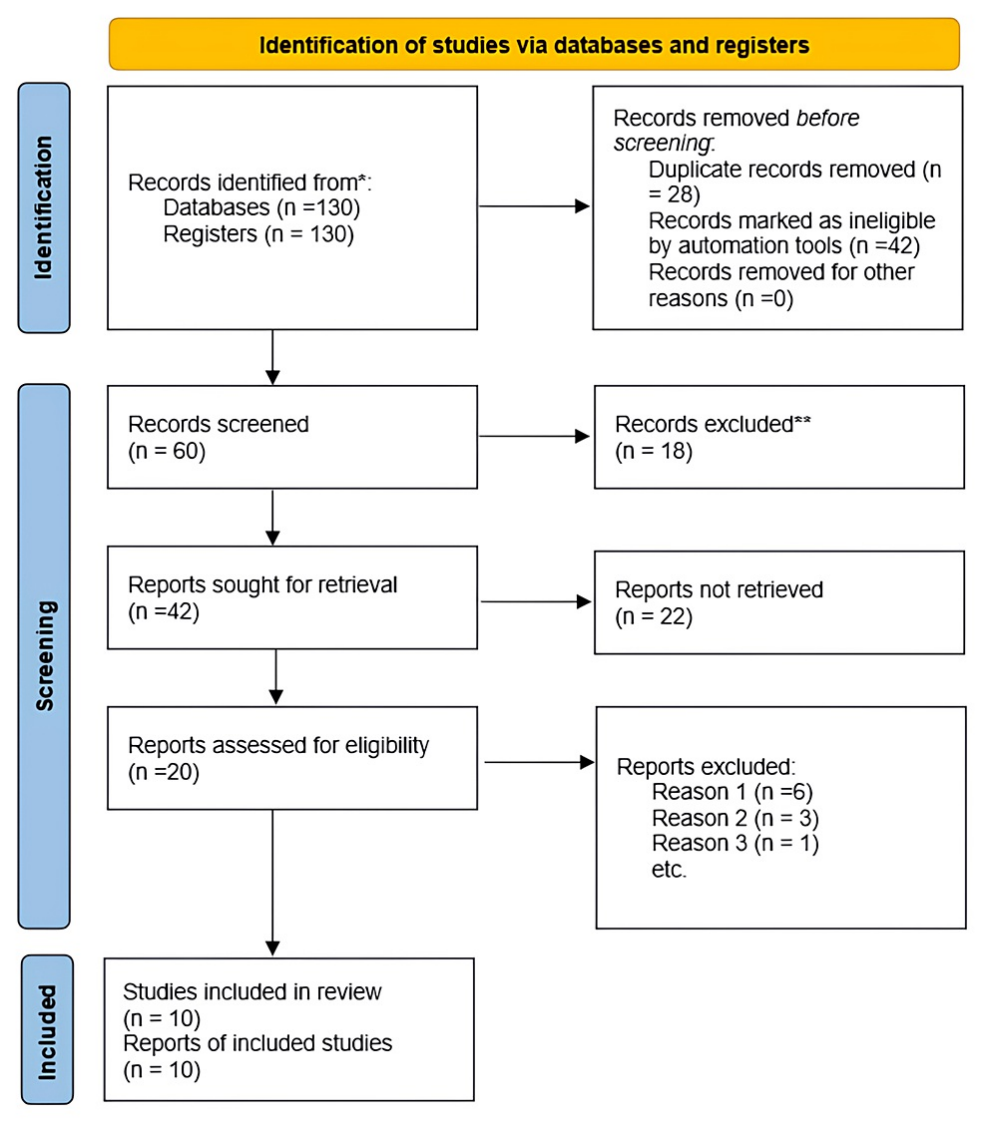
PRISMA chart

A thorough search of English-language literature in computerized databases was conducted using a methodical approach. The co-authors reached a consensus on the search. From 2012 to 2024, four distinct databases were searched: PubMed, Physiotherapy Evidence Database, Semantic Scholar, and Google Scholar. As a secondary search, the reference lists of the included research and relevant review papers were screened. Figure 1 illustrates the ten articles that were available for review.

Aetiology

The abnormal build-up of fluid in the pleural space, known as pleural effusion, is quite frequent. Based on US registration data, it is predicted that between 400,000 and 500,000 people in Germany are affected by this illness annually (specific German numbers are not available). Its aetiology is rather diverse, ranging from relatively benign effusions associated with viral pleuritis to highly significant ones associated with congestive heart failure or malignancy in terms of prognosis. The one-year mortality rate for patients with a non-malignant pleural effusion ranges from 25% to 57% [13].

Pathophysiology

**Figure 2:**
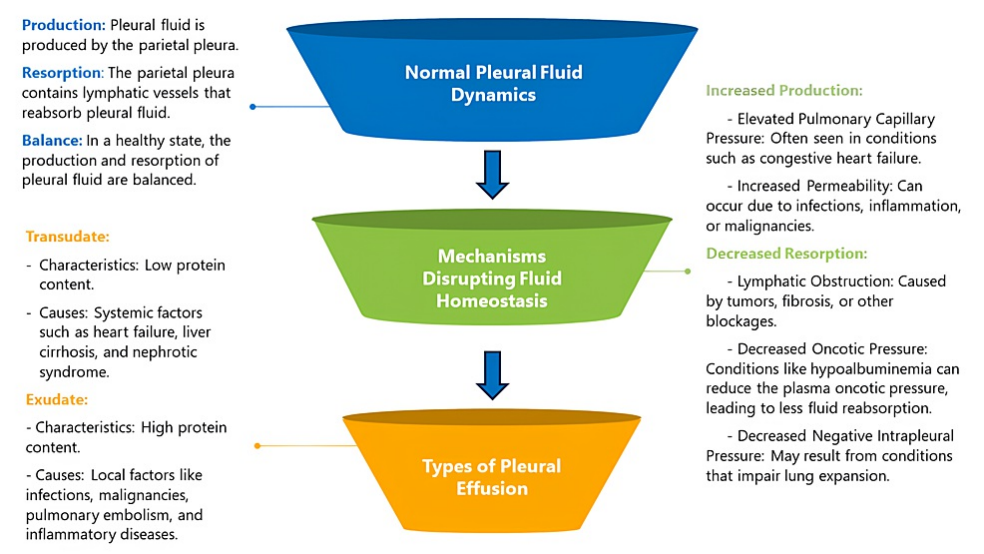
Flowchart of the pathophysiology of pleural effusion

The maintenance of fluid homeostasis in the pleural space is significantly influenced by the parietal and visceral pleura. There is an average rate at which the hydrostatic and oncotic pressure differences between the pleural space, the pulmonary circulation, and the systemic circulation are produced and absorbed. The parietal pleura contains lymphatic veins that reabsorb pleural fluid. The pleural lymphatic resorbing system has a considerable reserve capacity because if more pleural fluid is produced than usual, the flow in these vessels can rise by a factor of 20. Pleural fluid production and resorption are balanced in health. This equilibrium is upset by a pleural effusion, most likely as a result of both increased production and decreased resorption. The pathophysiological elements that result in the clinically significant and distinctive characteristics of a pleural effusion-transudate vs. -exudate include low oncotic pressure (such as in hypoalbuminemia), elevated pulmonary capillary pressure, increased permeability, lymphatic obstruction, and decreased negative intrapleural pressure [14]. Figure 2 shows a schematic flowchart.

Clinical history

The clinical history is crucial once it has been determined that there is either a unilateral or bilateral pleural effusion. It is important to enquire about fever, weight loss, malaise, and recent respiratory illnesses from the patient. Additionally, the temporal course is quite important: Did the symptoms appear suddenly or did they develop gradually, maybe over a few weeks? What additional long-term conditions does the patient have? Given that the most prevalent cause of bilateral pleural effusion is congestive heart failure, any history of heart illness must be disclosed. Pleuritic chest pain is reported by about 75% of the individuals with pleural effusion and pulmonary embolism [15].

Pathological diagnosis

MPE is brought on by tumour metastasis, which can happen by direct invasion, hematogenous dissemination, or lymphangitic spread into the pleura or pleural space. Tumour cells in the pleural fluid or proof of tumour presence in a pleural biopsy are necessary for the diagnosis of MPE. The diagnostic yield of a sonography-guided thoracentesis is 60% after the first aspiration and rises to 70-75% after the second aspiration, but then plateaus. The underlying type of tumour determines the sensitivity. Even less sensitive than the mentioned average is pleural fluid in the detection. Compared to image-guided (CT or USG) biopsy, which can have a yield as high as thoracoscopic parietal pleural biopsy (95%), blind percutaneous pleural biopsy is less sensitive. Video-assisted thoracoscopy (VATS) or medical thoracoscopy can be used to perform a thoracicoscopic biopsy. By Light's standards, the pleural fluid is typically exudative. Light's criteria are useful in identifying the cause of the pleural effusion as well as in differentiating between an exudative effusion and a transudate. If an effusion satisfies any of the following requirements, it is classified as exudative: Pleural fluid lactate dehydrogenase (LDH) to serum LDH ratio is larger than 0.6, pleural fluid protein to serum protein ratio is greater than 0.5, and pleural fluid LDH is greater than two-thirds of the upper limit of normal serum LDH. According to reports, 60 mL of pleural fluid is typically sufficient for diagnosis. However, since many of them do not show sufficient cellularity for genomic research, the use of pleural fluid for molecular sequencing is dubious, particularly in the treatment of lung cancer. This situation is probably going to be altered in the future as molecular and tumour marker testing continues to advance [16].

Table 1 shows data matrix.

**Table 1:** Data matrix RCT: randomized controlled trial; ABG: arterial blood gases; GCS: Glasgow coma scale; ICU: intensive care unit; POD: post-operative day; FVC: forced vital capacity; PEF: peak expiratory flow; FEV1: forced expiratory volume; VAS: visual analogue scale; ICD: intercostal drainage; CPAP: continuous positive airway pressure; TEN: transcutaneous electrical nerve stimulation.

Author and Year of Publication	Study Type	Study Sample	Intervention	Outcome Measure	Conclusion
Lester et al. 2022 [13]	RCT	61	Manometry-guided therapeutic thoracentesis for complaints of pleural effusions with lung expansion exercises	Radiography and visceral pleural recoil	Thoracic ultrasonography that shows radiographic lung re-expansion is not a good proxy for typical terminal pleural elastance. Future clinical trials should look at the clinical management of patients with recurrent symptoms in pleural effusions, which are guided by manometry rather than post-thoracentesis imaging, resulting in better outcomes.
Amal Abd El-Nasser Mohamed et al. 2021 [17]	RCT	60	The patient has been treated with one of the lung expansion techniques, i.e., stacked breathing exercises, which shows effectiveness in mobilizing a greater lung volume.	Chest examination, radiograph, ABG analysis, and haemodynamics (temperature, heart rate, respiratory rate, central venous pressure, and GSC)	Based on the protocol that was given, it was seen that keeping the patients in an adequate amount of hydration state and blocking the intravenous fluids had the effect of reducing the complications of pulmonary infection in pleural effusion patients.
Volodymyr Vitomskyi et al. 2020 [18]	Retrospective analysis	351	One of the practices for early mobilization patients was able to sit with their legs outside the bed during their ICU stay (usually on the first postoperative day); the second was to stand and walk on the spot within the ward within two post-operative days, and the third was to walk in the hospital corridor. Extra early mobilization patients used the first POD to practice sitting with their legs hanging off the side, standing with assistance and under physiotherapist supervision, holding onto a medical moveable walker with an anaesthesiologist’s permission, and, if practical, on-the-spot walking. Two options include walking on-the-spot and walking.	Ultrasound findings to check the fluids present in the spaces	Finally, the study concludes that there are no significant changes in early mobilization or extra-easily mobilization of patients with pleural effusion.
Hongwei Sun et al. 2020 [19]	RCT	400	All the patients were treated with unilateral lobectomy and were taught with lip retraction breathing and abdominal breathing. Also, after the operative procedure, three-ball breathing training was used in the observation group.	Drainage volume, FVC, PEF, FEV in the first, second FEV1 and FEV1/FVC	Properly and scientifically approved respiratory training helps to increase drainage flow and is also used to improve pulmonary functions in patients who underwent closed thoracic surgery after pleural effusion.
Parmar et al. 2019 [20]	RCT	40	Participants were divided into two groups: the traditional physiotherapy group, in which pursed lip breathing, segmental breathing, and active mobilization were given, and in the control group, transcutaneous electrical nerve stimulation was given.	Pain and dyspnoea on the VAS and chest expansion	This study was done on patients with ICD and saw that TEN, along with traditional physiotherapy, has a significant effect in reducing pain and dyspnoea and increasing chest expansion in pleural effusion patients.
dos Santos et al. 2019 [21]	RCT	156	The control group received just sham-positive airway pressure (4 cm H2O). The experimental group received incentive spirometry, airway clearance, mobilization, and the same sham-positive pressure.	Up until hospital release, data on chest tube drainage days, hospital stay duration, pulmonary problems, and adverse events were documented.	Positive pressure added to mobilization and respiratory treatments reduced hospital stays, length of thoracic drainage, pulmonary problems, use of antibiotics, and treatment expenses in pleural effusion patients.
Obaya et al. 2018 [22]	RCT	10	Patient was asked to lie in a supine position and the CPAP was generated with portable device and applied with the help of face mask and the pressure was sustained for 10 min. The image was captured with the help of deep breathing exercises with apnoea.	Computed tomography, tolerance level on analogue scale.	The study concluded that the CPAP with 15 cm H2O can expand lungs with pleural drainage and also shows that it is well tolerated and safe.
Gunjal et al. (2015) [23]	Prospective comparative study	30	In the first group, the patient is given deep breathing exercises, and in the second group, a segmental breathing exercise was recommended.	Chest expansion and pulmonary functions	After two weeks of physiotherapy sessions in which segmental breathing was given in one group, it was found that there were more effective results of chest expansion than compared to deep breathing exercises.
Valenza-Demet et al. (2014) [24]	RCT	104	The control group was treated with medicine and drainage. Intervention groups include standard medical and physiotherapy treatment, which includes deep breathing exercises, pursed lip breathing exercises, incentive spirometry, and active and passive mobilization techniques.	Major outcomes included in the study were chest radiographs before and after the treatment, values of spirometry before and after, and the total length of hospital stay of the patients.	The physiotherapy program (deep breathing, pursed lip breathing exercises, incentive spirometry and active and passive mobilization techniques) with standardized medical treatment that was given showed improvement in a chest radiograph, an increase in the amount of spirometry, and reduced hospital stay of the patients with pleural effusion.

Discussion

MPE occurs when cancer cells invade the pleura, leading to the accumulation of fluid in the pleural cavity [25]. Common cancers associated with MPE include lung cancer, breast cancer, and mesothelioma [26]. Managing MPE requires a multi-disciplinary approach, and physiotherapy plays a crucial role in addressing the respiratory complications associated with this condition. MPE can cause significant symptoms such as dyspnoea, chest pain, and reduced exercise tolerance [27]. Physiotherapists focus on symptom management through breathing exercises, pain management strategies, and techniques to improve overall functional capacity. Respiratory muscle weakness is cancer-related fatigue, and the direct impact of malignant cells on respiratory muscles can lead to muscle weakness [28]. Physiotherapy interventions include respiratory muscle training to improve strength and endurance, ultimately enhancing the patient's ability to cope with respiratory distress [29]. The goal of physiotherapy in MPE is to improve the patient's quality of life. This involves addressing both the physical and psychological aspects of symptom burden. Tailored exercise programmes can contribute to increased mobility, reduced fatigue, and improved mental well-being [30]. Physiotherapists educate patients about breathing techniques, energy conservation strategies, and the importance of maintaining physical activity. This empowerment helps patients actively participate in their care and cope with the challenges associated with MPE [31].

Devices, such as incentive spirometer and positive expiratory pressure (PEP) devices, are valuable in the management of MPE to address respiratory symptoms and improve lung function. Incentive spirometers in patients with MPE and incentive spirometry can aid in maintaining lung expansion, preventing atelectasis, and promoting optimal lung function. Regular use of an incentive spirometer helps patients achieve deeper breaths, which is crucial for managing respiratory symptoms [32]. PEP devices are beneficial for patients with MPE by providing positive pressure during expiration. This can assist in keeping airways open, improving lung compliance, and facilitating the clearance of secretions. PEP therapy can be integrated into the overall respiratory care plan for enhanced effectiveness [33]. Palliative care integration in cases where the disease is advanced and curative treatments may not be possible, physiotherapy, including the use of an incentive spirometer and PEP, is an essential component of palliative care [34]. The focus shifts towards symptom control, maintaining functional independence, and enhancing the overall quality of life. In conclusion, the integration of lung boost devices into physiotherapy interventions for MPE is aimed at improving respiratory function, managing symptoms, and enhancing the overall well-being of patients facing the challenges of advanced cancer-related pleural complications [34].

An randomized control trial (RCT) was undertaken by Gunjal et al. on the effectiveness of sustained maximal inspiration along with transcutaneous electrical nerve stimulation in MPE with intercoastal drainage. The 44 patients were divided into two groups: the control group was given medical treatment along with sustained maximal stretch, and the experimental group was given the same as the control group with the addition of transcutaneous electrical nerve stimulator. The study concluded that the combined effect improves functions and reduces pain in patients of MPE with ICD [23]. The study showed that patients collected fluids in the pleural spaces and the hospital stay, pulmonary complications, and thoracic drainage decreased with respiratory techniques and positive pressure to mobilization. Overall 156 patients were taken into consideration and the treatment given was positive airway pressure with incentive spirometry and mobilisation [20].

Mohamed et al., in their study, concluded that patients were given a stacked breathing technique in one group, and in another group, it was given with normal hospital care. A total of 60 patients were taken into consideration, and it was seen that the stacked breathing technique is more effective in treating patients with pleural effusion [17]. Sun et al. conducted a study on the short-term effects of respiratory function training on patients with pleural effusion undergoing closed drainage and related impact on pulmonary function. In this study, the major focus was on the drainage volume and pulmonary functions with the intervention given as lip retraction breathing, abdominal breathing technique, and three-ball breathing technique, which all help in increased drainage flow and pulmonary functions [19].

This study shows us that the physiotherapy protocol administered to the pleural effusion patients gives an effective result. Physiotherapy is most useful for pleural effusion in chest expansion, and deep breathing exercises are most important for pleural effusion patients. This comprehensive review on the effectiveness of physiotherapy interventions in pleural effusion patients has several limitations, including a limited number of studies, variability in intervention types, and differences in patient characteristics, which affect the generalizability of the findings. Additionally, the lack of high-quality randomized controlled trials and the potential for publication bias further highlight the need for more rigorous research in this area.

## Conclusions

Thus, the pleural effusion rehabilitation involving conventional chest physiotherapy, which includes various breathing techniques and lung expansion techniques, has proven to be more effective in enhancing lung volume capacity in patients. These interventions not only aid in improving lung function but also contribute to better oxygenation and overall respiratory health. Other studies have demonstrated the effectiveness of various protocols in treating pleural effusion, yet they consistently highlight the central role of breathing exercises and lung expansion techniques. These protocols focus on strengthening the respiratory muscles, facilitating better fluid drainage, and promoting overall respiratory efficiency, underscoring the importance of tailored physiotherapy interventions in the comprehensive management of pleural effusion.
